# Synthesis of compounds based on the active domain of cabotegravir and their application in inhibiting tumor cells activity

**DOI:** 10.1002/open.202300284

**Published:** 2024-02-05

**Authors:** Ruyue Yang, Wenhui Yue, Dong Hu, Guidan Wang, Longfei Mao, Jiahe Huang, Huili Wang, Gaofeng Liang

**Affiliations:** ^1^ School of Basic Medicine and Forensic Medicine Henan University of Science & Technology Luoyang 471023 China; ^2^ School of Medical Technology and Engineering Henan University of Science & Technology Luoyang 471023 China; ^3^ University of North Carolina Hospitals 101 Manning Dr Chapel Hill Orange County NC 27599 USA

**Keywords:** Cabotegravir, derivatives, ESCC, KYSE30, apoptosis

## Abstract

Structural modification based on existing drugs, which ensures the safety of marketed drugs, is an essential approach in developing new drugs. In this study, we modified the structure of cabotegravir by introducing the front alkyne on the core structure through chemical reaction, resulting in the synthesis of 9 compounds resembling 1,2,3‐triazoles. The potential of these new cabotegravir derivatives as tumor suppressors in gastrointestinal tumors was investigated. Based on the MTT experiment, most compounds showed a reduction in the viability of KYSE30 and HCT116 cells. Notably, derivatives 5b and 5h exhibited the most significant inhibitory effects. To further explore the effects of derivatives 5b and 5h on gastrointestinal tumors, KYSE30 cells were chosen as a representative cell line. Both derivatives can effectively curtail the migration and invasion capabilities of KYSE30 cells and induce apoptosis in a dose‐dependent manner. We further demonstrated these derivatives induce cell apoptosis in KYSE30 cells by inhibiting the expression of Stat3 protein and Smad2/3 protein. Based on the above results, we suggest they show promise in developing drugs for esophageal squamous cell carcinoma.

## Graphical abstract

The objective of this study was to assess the inhibitory effect of modified cabotegravir derivatives on the activity of esophageal cancer KYSE30 cells. Results showed that compounds 5b and 5h effectively inhibited the migration and invasion of KYSE30 cells and induced apoptosis and may be involved in the stat3 and smad2/3 signal pathway. Based on these findings, we can consider these compounds as potential lead candidates for the treatment of esophageal squamous cell carcinoma.

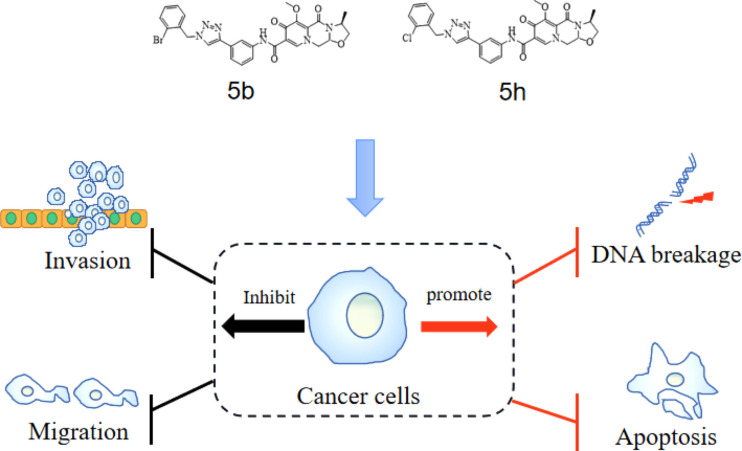

## Introduction

The global incidence rates of esophageal cancer and colon cancer are high and continue to rise.[^1^, ^2^] According to statistics from 2020, esophageal cancer ranked ninth and colon cancer ranked fifth among malignant tumors in terms of incidence rate. Additionally, the mortality rate for esophageal cancer ranked sixth and for colon cancer ranked fifth.^[3]^ These two types of cancer pose a significant threat to human health.^[4]^ Existing treatment methods for tumors include surgery, radiotherapy, chemotherapy, and immunotherapy.[^5^, ^6^] However, these methods have various limitations, for instance, surgery may not completely eradicate the tumor, and there are risks of potential complications. Additionally, systemic toxicity, low tumor specificity, and drug resistance are common challenges associated with these treatments.[^7^, ^8^] As a result, overall survival rates remain low.^[9]^ Therefore, there is an urgent need to develop more effective and targeted treatment options.

Chemotherapy plays an essential role in treating ESCC,^[10]^ the combination of 5‐fluorouracil plus cisplatin or cisplatin plus paclitaxel is commonly used in chemotherapy.[^11^, ^12^] In a study by Wang Hao et al., Combining the chemotherapy drugs paclitaxel and cisplatin with a radiation dose of 40 Gy in esophageal cancer patients led to higher rates of pathologic complete response and negative lymph nodes.^[13]^ According to Tong, L. et al., the combination of 5‐fluorouracil and oxaliplatin can effectively decrease the risk of mortality and enhance overall survival rates.^[14]^ These findings emphasize the crucial role of chemotherapy in the comprehensive treatment of cancer. However, cisplatin use is often associated with significant toxicity, such as gastrointestinal adverse reactions, kidney damage, and neurotoxicity.[^15^, ^16^] The effectiveness of first‐line chemotherapy for ESCC using 5‐fluorouracil, cisplatin, and paclitaxel is limited, resulting in a less than 20 % 5‐year survival rate for patients with advanced esophageal cancer.[^17^, ^18^] Out of the situation mentioned above, improving the antitumor efficacy of chemotherapy while minimizing toxic side effects and enhancing patients′ overall prognosis and quality of life remains a critical challenge in developing chemotherapeutic drugs for cancer.

Currently, many chemotherapy drugs available in the market have numerous adverse reactions, which pose challenges for patients in tolerating them and can ultimately lead to adverse outcomes.^[19]^ The research and development of antitumor drugs is an immensely challenging task that holds significant importance in life sciences today. Structural modification based on existing drugs, which ensures the safety of marketed drugs, is an essential approach to developing new drugs.^[20]^ Cabotegravir (CAB) is a long‐acting HIV‐1 integrase strand transfer inhibitor that prevents the integration of viral DNA into the genome of human immune cells.[^21^, ^22^] In HIV prevention (PrEP), cabotegravir is being investigated as a potential monotherapy and is considered an excellent candidate for PrEP due to its extended half‐life, allowing for less frequent dosing.^[23]^ Cabotegravir is an attractive option for those with difficulty adhering to other prevention methods.[^24^, ^25^] Current research on cabotegravir primarily focuses on its anti‐HIV properties. It does not indicate its potential application in treating other diseases.

In previous study, our group conducted modifications on icotinib and successfully synthesized its 1,2,3‐triazole derivative. Several of these compounds exhibited remarkable antitumor activity than icotinib against one or more cancer cell lines, and the results suggested a possible induced NSCLC cells death via inducing mitochondrial apoptosis and arresting cell cycle^[26]^ Inspired by the above results, in order to explore the potential of cabotegravir in cancer treatment, we synthesized nine structurally novel cabotegravir derivatives (5a–5i) while reserving the active domain of cabotegravir. The activity of these compounds on KYSE30 and HCT116 cells was evaluated using the MTT assay, and two compounds (5b and 5h) were selected after screening. Further evaluation was conducted to assess their effects on tumor cell migration, invasion, and apoptosis. The results demonstrated that these compounds inhibited cell proliferation and suppressed migration and invasion abilities. Western blot analysis also indicated that the compounds may be involved in the apoptosis pathway of cells, resulting from a decrease in the expression of Stat3 protein and Smad2/3 and an increase in the expression of the apoptosis‐related protein cleaved caspase 3. Based on our analysis, it is evident that synthetic cabotegravir derivatives show promise in the field of oncology drug development.

## Results and Discussion

### Characterization of compounds

**Scheme 1:**
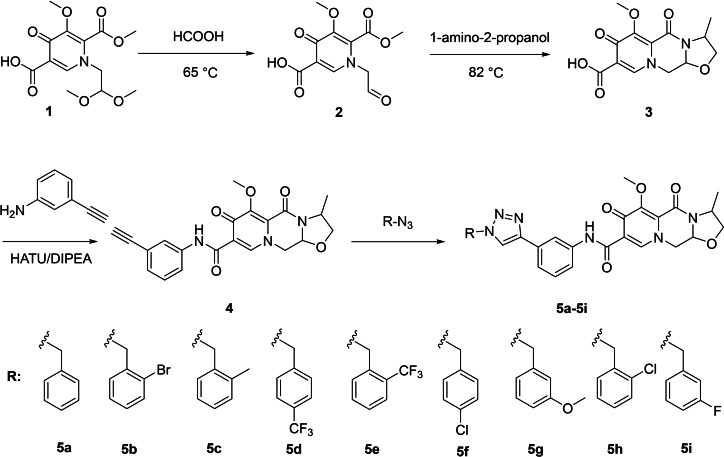
The reaction routes to compounds 5a–5i.

The respective structures of these newly synthesized compounds were elucidated and documented in Scheme 1. All synthesized compounds were purified using column chromatography, followed by comprehensive characterization through spectral analyses, including ^1^H NMR, ^13^C NMR, and MS techniques. Further detailed information concerning the ^1^H NMR and ^13^C NMR spectra can be found in the supplementary files.

### Compounds 5a‐5i suppress the viability of KYSE30 and HCT116 cells

To investigate the inhibitory activity of these synthesized derivatives on gastrointestinal cancer, we selected KYSE30 and HCT116 cells for cell viability assay. The results are shown in Table 1. Based on the MTT results, it was found that compounds 5b and 5h exhibited the most effective killing effect on cancer cells, the IC_50_ values against KYSE30 cells were (5.15±1.55) and (7.13±0.91) μM, while against HCT116 cells, the IC_50_ values were (4.14±1.67) and (8.90±2.87) μM. To further investigate the impact of compounds 5b and 5h on cancer, KYSE30 cell line was selected as a representative cell line for conducting subsequent invasion, migration, apoptosis, and western blot assays.

**Table 1:** The half‐maximal inhibitory concentration (IC_50_) of some compounds.

Compd no.	KYSE30	Compd no.	KYSE30	Compd no.	HCT116	Compd no.	HCT116
	IC_50_ (μM)		IC_50_ (μM)		IC50 (μM)		IC50 (μM)
5a	24.96±5.58	5 g	34.81±3.43	5a	32.58±5.07	5g	54.46±7.12
5b	5.15±1.55	5 h	7.13±0.91	5b	4.14±1.67	5h	8.90±2.87
5c	12.02±1.84	5i	35.88±7.47	5c	14.15±5.03	5i	31.23±2.87
5d	12.60±1.73	Cisplatin	16.95±2.56	5d	14.11±4.21	5‐ Fluoruouracil	22.88±0.14
5e	8.75±0.57	Cabotegravir	>50	5e	13.68±4.51	Cabotegravir	>50
5f	20.53±4.63			5f	>50		

### Effect of 5 b and 5 h on the migration of KYSE30 cells

**Figure 1:**
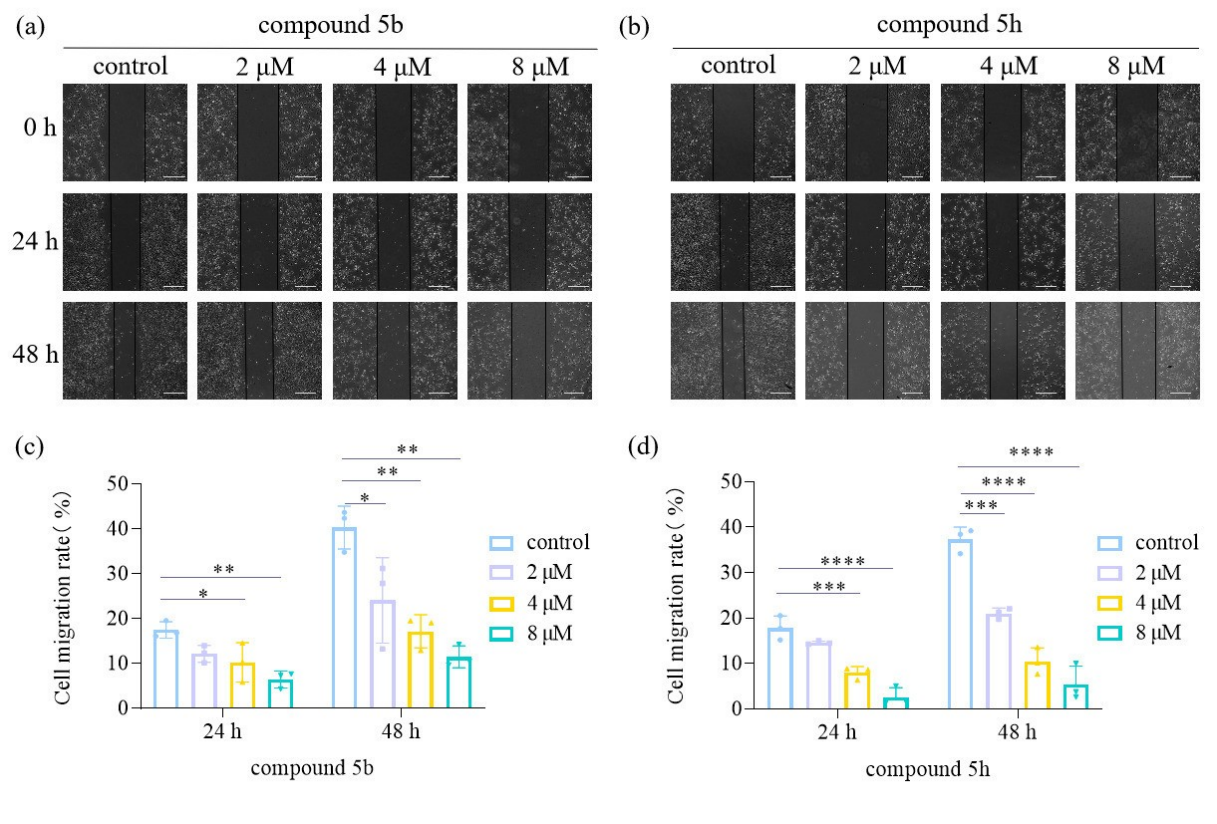
Compounds 5b and 5 h inhibited the migration ability of KYSE30 cells. Photographs of cells at 0 h, 24 h and 48 h after treatment with different concentrations of 5b (a) and 5 h (b). (Scale bar=200 μm). (c): Effect of 5b on the migration rate of KYSE30 cells. (d): Effect of 5 h on the migration rate of YSE30 cells (n=3, *P<0.05). Data are shown as mean±SD and analyzed by one‐way ANOVA. (*P<0.05, **P<0.01, ****P<0.0001 compared to control group).

In the migration experiment, the change in scratch width serves as an indicator of cell migration ability. Different groups of cells were exposed to drug concentrations of 2 μM, 4 μM, and 8 μM or 0.1 % DMSO (as control). The change in scratch width was recorded by photographing at 0 hours, 24 hours, and 48 hours. The cell migration rate was calculated using the formula: cell migration rate=(mean initial intercellular distance ‐ mean intercellular length at time t) / mean initial intercellular distance. After adding compound 5b for 24 hours, the cell migration rates for each group were as follows: 17.50 % (control), 12.18 % (2 μM), 10.28 % (4 μM), and 6.45 % (8 μM). After 48 hours, the cell migration rates for each group were 40.30 % (control), 24.09 % (2 μM), 17.19 % (4 μM), and 11.50 % (8 μM). Compared to the control group, the experimental groups exhibited a gradual decrease in cell migration ability with increasing drug concentration (Figure 1). Similarly, when different concentrations of compound 5 h were applied to KYSE30 cells, the cell migration rates after 24 hours were: 17.81 % (control), 14.59 % (2 μM), 7.97 % (4 μM), and 2.52 % (8 μM). After 48 hours, the cell migration rates were 37.29 % (control), 21.02 % (2 μM), 10.47 % (4 μM), and 5.45 % (8 μM). These findings demonstrate that 5 h also led to a decrease in cell migration ability in a dose‐dependent manner.

### Effect of 5 b and 5 h on the invasive ability of KYSE30 cells

**Figure 2:**
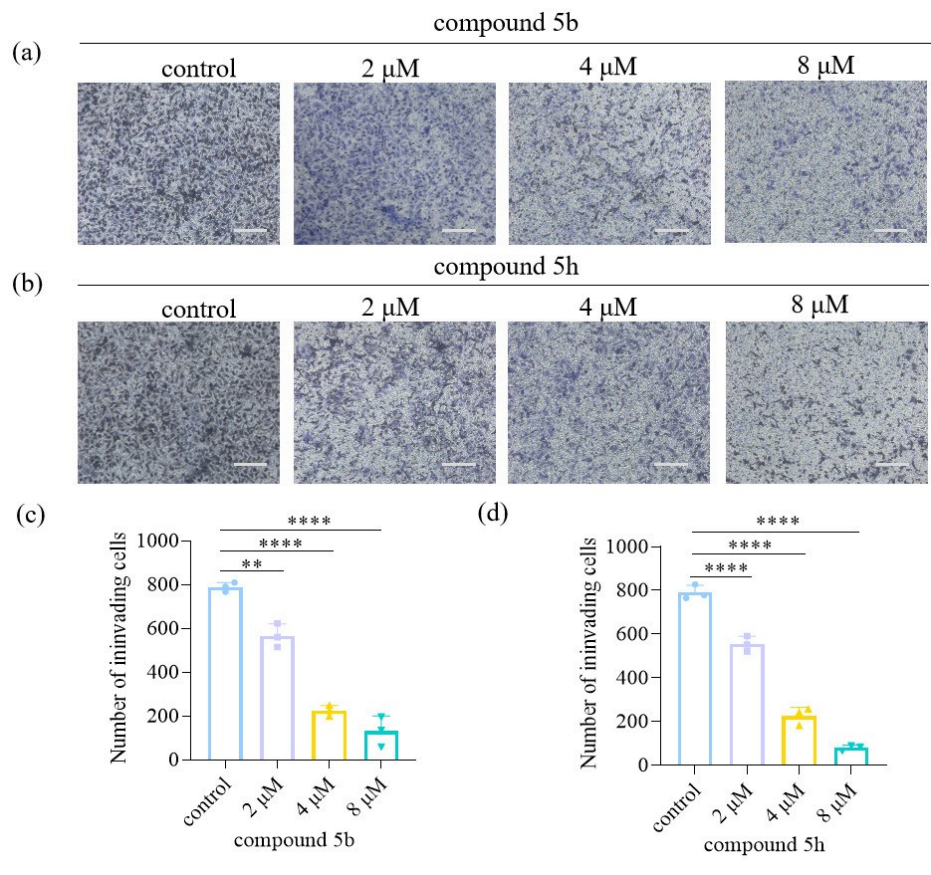
Effect of 5b and 5 h on the invasive ability of KYSE30 cells. (a)(b): Using microscopy to record cell invasion after cells were treated with different concentrations of compounds for 24 h. (Scale bar=200 μm). Effects of different concentrations of 5b (c) and 5 h (d) on invasion rate of KYSE30 cells (n=3). Data are shown as mean±SD and analyzed by one‐way ANOVA. (*P<0.05, **P<0.01, ****P<0.0001 compared to control group).

To investigate the effect of compounds 5b and 5 h on the invasive ability of cells, transwell experiments were performed, and the results are presented in Figure 2. Transwell assay results indicated that, compared with the control group (0.1 % DMSO), both 5b and 5 h affected the invasive ability of KYSE30 cells. Moreover, the invasive ability of the cells decreased progressively with increasing drug concentration. These findings suggest that 5b and 5 h can inhibit the invasion of esophageal cancer cells.

### Effects of 5 b and 5 h on apoptosis of KYSE30 cells

**Figure 3:**
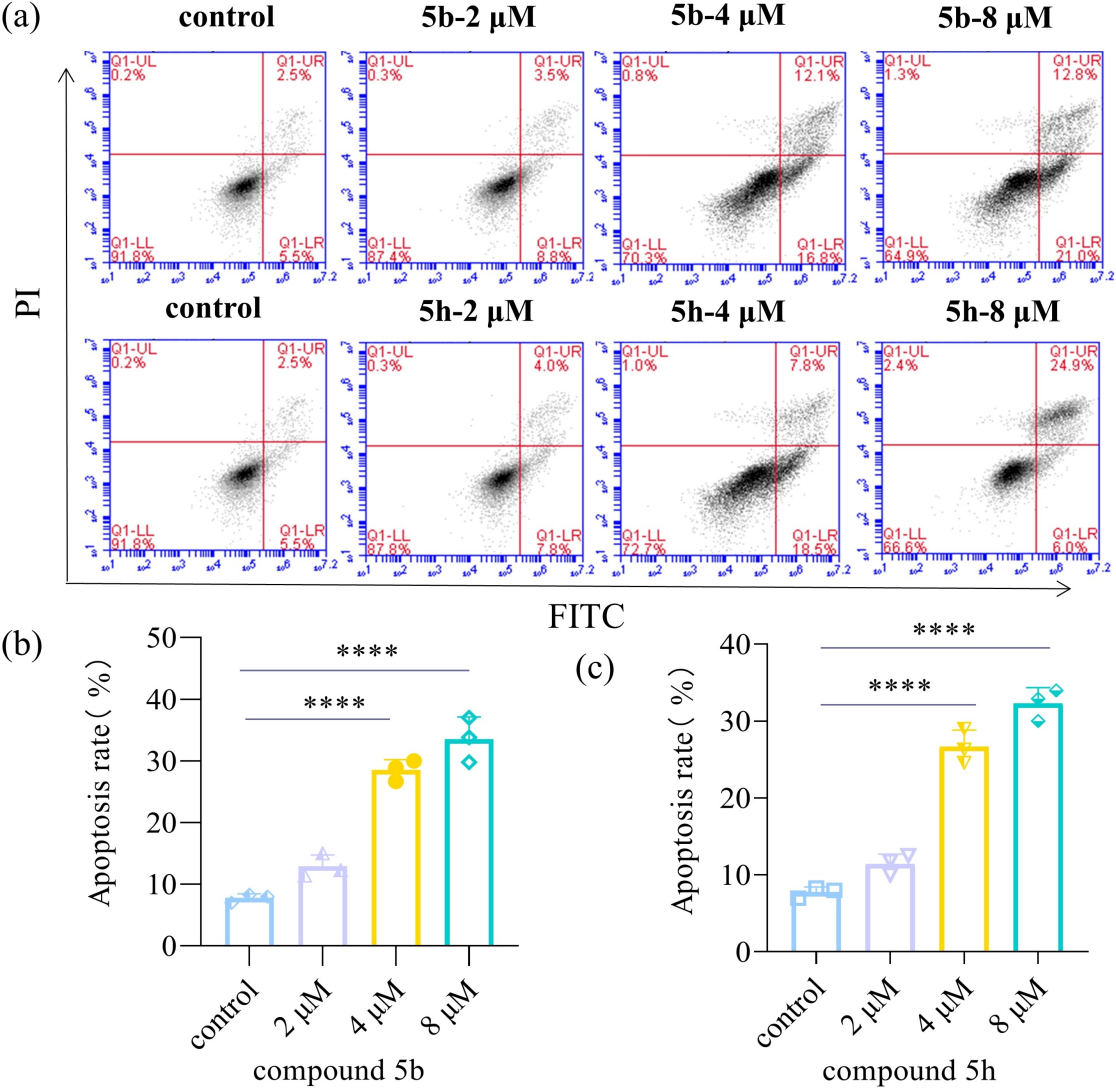
Effects of 5b and 5 h on apoptosis of KYSE30 cells. (a): Apoptosis flow results. (b): Effect of different concentrations of 5b on apoptosis of KYSE30 cells (n=3). (c): Effect of different concentrations of 5 h on apoptosis of KYSE30 cell(n=3). Data are shown as the mean±SD and were analyzed by one‐way ANOVA. (*P<0.05, **P<0.01 and ****P<0.0001, compared with the control group).

**Figure 4:**
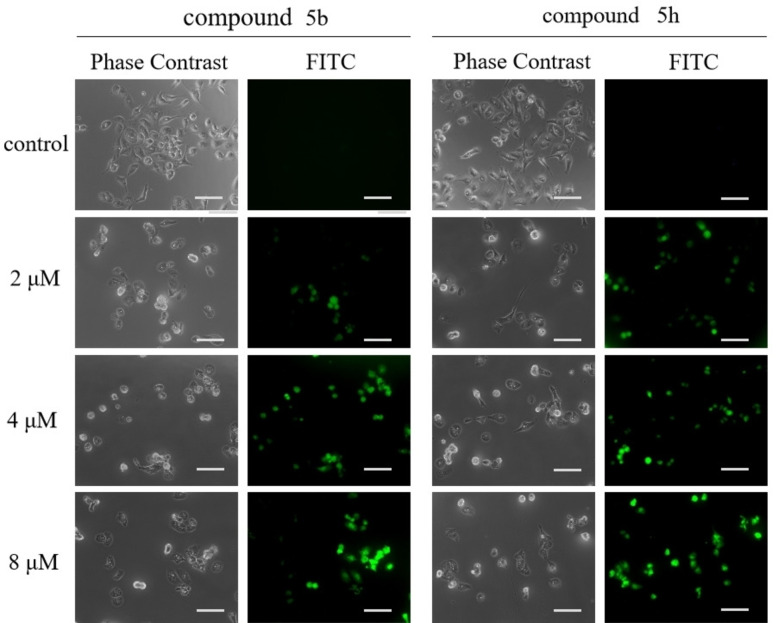
TUNEL staining to observe the effect of different drugs on DNA breakage in KYSE30 cells. (Scale bar =100 μm).

Relevant experiments were conducted to determine whether the inhibitory and cytotoxic effects of 5b and 5 h are associated with cell apoptosis. The control group was treated with 0.1 % DMSO, while the experimental group was treated with different concentrations of the compounds. After 48 hours, the cells were stained with Annexin V‐FITC and PI, and the percentage of apoptotic cells was measured using flow cytometry. As depicted in Figures 3B and 3 C, the proportions of KYSE30 apoptotic cells treated with 5b were 12.3 % (2 μM), 28.9 % (4 μM), and 33.9 % (8 μM) compared to the control group. Similarly, the proportions of KYSE30 apoptotic cells treated with 5 h were 11.8 % (2 μM), 26.3 % (4 μM) and 30.9 % (8 μM). These results demonstrate that 5b and 5 h significantly promote apoptosis in KYSE30 cells and positively correlate with the drug dose. Furthermore, the TUNEL assay were conducted to verify the effect of the compound on DNA breakage in cells, and the result is shown in Figure 4. After being treated with different concentrations (2, 4, 8 μM) of 5b and 5 h, the fluorescence levels increased proportionally to the drug concentration. This result indicates a gradual intensification of DNA fragmentation in the cells. Flow cytometry and TUNEL experiments demonstrated that compounds 5b and 5 h effectively induced apoptosis in KYSE30 cells.

### Investigation on the mechanism of 5 b and 5 h induced apoptosis of KYSE30 cells

**Figure 5:**
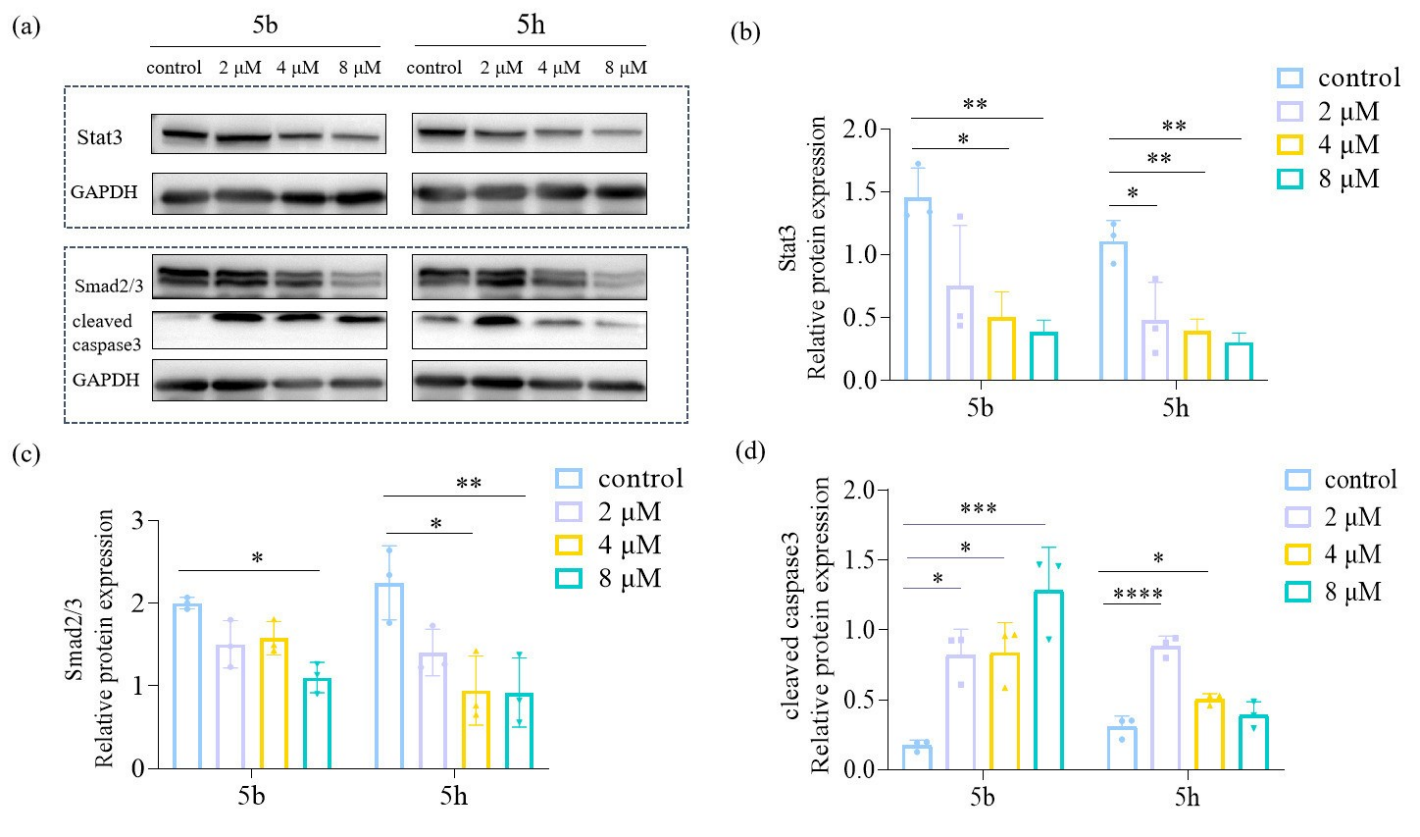
(a): Western blot was used to detect the protein expression of KYSE30 cells treated with different concentrations of compounds. (b): Western blot results showed that the expression of Stat3 protein was significantly decreased after treatment with different concentrations of the compounds. (c): The expression of Smad2/3 protein was significantly decreased after treatment with different concentrations of drugs. (d): The expression of cleaved caspase3 protein was significantly increased after treatment with different drug concentrations. Data are shown as the mean±SD and were analyzed with one‐way ANOVA. (*P<0.05, **P<0.01, ***P<0.001, and ****P<0.0001, compared with the control group).

As a transcription factor, Signal Transducer and Activator of Transcription 3 (Stat3) regulates various genes involved in cancer cell survival, proliferation, angiogenesis, invasion, metastasis, drug resistance, and immune evasion.^[27]^ It is often highly activated in multiple tumor types, including esophageal squamous carcinoma cells.^[28]^ Overexpression and sustained activation of Stat3 have been linked to esophageal squamous carcinogenesis.^[29]^ Our study aimed to investigate the impact of 5b and 5 h on Stat3 in KYSE30 cells. Through Western blotting experiments, we observed that both 5b and 5 h could down‐ regulate Stat3 expression. (Figure 5B). Besides, to further understand the mechanistic pathway through which 5b and 5 h affect KYSE30 cell apoptosis, we examined the expression of

Smad2/3 proteins and cleaved caspase 3 protein, which indicates apoptosis in KYSE30 cells. The results are shown in Figure 5C and Figure 5D. Western blot results showed that after treatment with different concentrations of drugs, Stat3 and Smad2/3 protein expression decreased while cleaved caspase3 expression increased compared with that of the control group. This phenomenon is due to Stat3 activation associated with the Smad complex‘s nuclear localization, which subsequently promotes epithelial‐mesenchymal transition (EMT).^[30]^ Thus, by downregulating the Stat3 protein, these compounds may inhibit EMT in KYSE30 cells. The results suggest that the drug may inhibit the biological function of KYSE30 cells by Stat3 and Smad2/3‐related pathways. Observing an elevation in cleaved caspase 3 protein further supports the notion that 5b and 5 h can induce apoptosis in KYSE30 cells. Our findings suggest that 5b and 5 h affect KYSE30 cells by inducing apoptosis.

## Conclusions

We employed a method that involves replacing hydroxyl groups with methoxy groups in the active domain of cabotegravir. We successfully obtained a series of structurally novel compounds through click reactions. These nine newly synthesized compounds were evaluated for their ability to inhibit tumor cell growth. MTT experiments were conducted to verify their inhibitory effects, and most compounds demonstrated significant inhibitory effects. Among them, compounds 5b and 5h exhibited the highest inhibitory effects. Furthermore, we observed that compounds 5b and 5h induced apoptosis in KYSE30 cells, promoted DNA fragmentation, and participated in the apoptosis pathway. Among these compounds, the best anti‐tumor effect is observed when the halogen atoms are present (compounds 5b and 5h) at the ortho‐position of the benzene ring, and the second‐best effect is observed when trifluoromethyl is present (compound 5e), while the anti‐tumor effect is weakest when hydrogen atom or methyl are present at the ortho‐positions. From the above fact, we know that the installation of halogen atoms (5b: Br, 5h: Cl, 5e: CF_3_) at the ortho‐positions of the benzene ring enhances the anti‐tumor effect of the corresponding compound. This enhanced anti‐tumor effect of compounds 5b, 5h, and 5e may be caused by the interaction between halogen atoms (possessing significant electronegativity) and targets in tumor cells. As for the fact that compound 5e has a more considerable IC_50_ value than compounds 5b and 5h, it may be due to the larger size of trifluoromethyl, which hinders the interaction of compound and target. Due to the limited number of synthesized compounds, conducting effective structure‐activity relationship studies proved challenging in this study. We have only had a preliminary discussion on this issue. In future research, we plan to explore more effective structure‐activity relationships by incorporating 1,2,3‐triazole structures into the para and ortho positions of the benzene ring that connects the core of cabotegravir.

## Experimental Section

### Materials and Chemistry

These derivatives, similar to 1, 2, 3‐triazole were synthesized in‐house using reagents and solvents obtained from a commercially available source. Compound 1 was purchased from Hubei CHU Sheng Wei Chemical Co. LTD(CAS:1335210‐23‐5). The ^1^H NMR and ^13^C NMR spectra were acquired in a DMSO‐d_6_ solution using a Bruker 400 MHz or 600 MHz NMR spectrometer. The primary antibodies of Stat3, Smad2/3, and cleaved caspase 3 were purchased from Cell Signaling TECHNOLOGY (CST, Massachusetts, USA), glyceraldehyde‐3‐phosphate dehydrogenase (GAPDH) and secondary antibodies were purchased from Shanghai Sangon Biotech (Sangon, Shanghai, China).

#### Synthesis of Compound 2

Compound 1, 1‐(2, 2‐dimethoxylethyl)‐1, 4‐dihydro‐3‐methoxy‐4‐oxo‐2, 5‐pyridine dicarboxylic acid‐2‐methyl ester (310 g, 0.98 mol), was added into anhydrous formic acid (1000 mL) and the solution was stirred at 65 °C for 3 h under an argon atmosphere, monitored by thin‐layer chromatography (TLC). After the reaction was completed, the solvent and formic acid were removed under reduced pressure to give a crude oily product. The resulting crude product was redissolved in acetonitrile, followed by concentration to remove formic acid again and generate the product (245 g, 93% yield).

#### Synthesis of Compound 3

Compound 2 (242.1 g, 0.90 mol) and 1‐Amino‐2‐propanol (105 g, 1.4 mol) were added into 1000 mL acetonitrile, and the solution mixture was stirred for 10 min. Then, the solution was heated to 80 °C and stirred for 6 hours. The solvent was then removed under reduced pressure. The residual was mixed with HCl (a.q) (pH 1–2), and the resulting solution was extracted with DCM three times. The organic solution was collected and washed twice with a saturated sodium chloride aqueous solution. The combined organic layer was dried over sodium sulfate and concentrated under reduced pressure to give the crude product. Finally, the crude product was further recrystallized using methanol to generate compound 3 (197 g, 74% yield).

#### Synthesis of Compound 4

Compound 3 (29.4 g, 0.1 mol), 3‐aminophenylacetylene (23.4 g, 0.2 mol), HATU (38 g, 0.1 mol), DIPEA (25.8 g, 0.2 mol) was added into 1500 mL DMF and the solution mixture was stirred at room temperature for 15 h under nitrogen protection. After the reaction was completed, the solvent was removed under reduced pressure. The residual was redissolved in dichloromethane and washed twice with a saturated sodium chloride aqueous solution. Then, the solution was dried over sodium sulfate, followed by the removal of the solvent under reduced pressure togenerate compound 4 (34.2 g, 87% yield).

#### General synthetic procedure for compounds 5a‐5i

Compound 4 (1.18 g, 3 mmol), substituted azide (3.6 mmol), TERT butanol (70 mL), H_2_O (70 mL), THF (70 mL), anhydrous copper sulfate (0.96 g, 6 mmol) and sodium ascorbate (0.2 g, 1 mmol) were successively added in the reaction flask. Then, the mixed solution was stirred and refluxed at 70 °C for 5 h. After the reaction was completed, the resulting solution was extracted with dichloromethane three times. The organic solution was collected and washed twice with a saturated sodium chloride aqueous solution. The combined organic layer was dried over sodium sulfate and concentrated under reduced pressure to give the crude product. Recrystallization of the resulting crude product in ethyl acetate produced the desired compound, which was pure enough for further characterization and anti‐tumor study.

The spectroscopic characterization of compounds 5a–5i is provided as Supporting Information Data.

#### Cell Culture

The KYSE30 and HCT116 cells were obtained from the Cell Bank of the Chinese Academy of Sciences in Shanghai, China. KYSE30 cells were cultured in Roswell Park Memorial Institute‐1640 medium (WISENT, Nanjing, China). HCT116 cells were cultured in Dulbecco′s Modified Eagle Medium (WISENT, Nanjing, China), In addition, 10 % fetal bovine serum (FBS), penicillin (100 U/mL), and streptomycin (100 mg/mL) were added. The cells were incubated at 37 °C in a 5 % CO_2_ environment.

#### Cell viability assay

We performed the Methyl thiazolyldiphenyl‐tetrazolium bromide (MTT) assay to evaluate cell viability. KYSE30 and HCT116 cells were seeded in a 96‐well plate at a density of 5×10^3^ cells per well and incubated for 24 hours at 37 °C under 5 % CO_2_ to allow cell attachment and growth. After 48 hours of compound treatment, 15 μL of MTT solution (5 mg/mL) was added to each well. The cells were then incubated for an additional 4 hours at 37 °C to allow the MTT reagent to be metabolized by viable cells. After incubation, the supernatant was carefully removed from each well, and 150 μL of dimethyl sulfoxide (DMSO) was added to dissolve the formed formazan crystals. The plates were shaken for 10 minutes to ensure complete dissolution of the crystals. A microplate reader (BioTek, Winooski, VT, USA) was utilized to measure the absorbance of the wells at 490 nm.

#### Wound‐healing assay

A wound‐healing assay was employed to evaluate the effect of compounds on the migration ability of KYSE30 cells. A total of 5×10^5^ KYSE30 cells were seeded in 6‐well plates. Once the cells reach 80 % confluence, a scratch or wound was created in the cell monolayer. After removing any detached cells and debris, a Serum‐free medium containing various concentrations of compounds was added to the plates, and the cells were incubated under appropriate conditions. The cells were photographed at 0 hours (immediately after creating the scratch), 24 hours, and 48 hours using a microscope. The degree of wound closure was then analyzed using Image J software from the National Institutes of Health in Bethesda, MD, USA.

#### Transwell assay

Before the experiment, Matrigel gel was diluted (50 mg/mL Matrigel gel/serum‐free 1640=1 : 8), then the diluted Matrigel gel was placed on top of the transwell chamber (ABW, shanghai, China). The chambers were allowed to stand at 37 °C for 3 hours to wait for film formation. Then, Esophageal squamous cell carcinoma cells were collected and mixed evenly with a serum‐free medium. A total of 2.5×10^4^ cells were added to the transwell chamber. In the next step, 500 μL of medium containing 10 % FBS was added to the bottom of a 24‐well plate. The plate was incubated in a 5 % CO_2_ incubator at 37 °C for 24 hours. After incubation, the cells were fixed with 4 % paraformaldehyde for 1 hour, stained with crystal violet for 20 minutes, and photographed under a microscope. Cells that invaded the lower chamber were then counted using Image J software.

#### Apoptosis assay

Cell apoptosis was detected by Annexin V‐FITC/PI Cell Apoptosis Detection Kit (Servivebio, China). KYSE30 cells with different treatments were collected and centrifuged at 1500 rpm for 5 minutes. The supernatant was carefully discarded, and the cell pellet was resuspended with phosphate‐buffered saline (PBS). Adding 100 μL binding buffer to the cells and gently mixing to prepare the cell suspension. Then, 5 μL of Annexin V‐FITC and 5 μL of propidium iodide (PI) were added to the cell suspension. The cell suspension was gently mixed and incubated at room temperature in the dark for 10 minutes. BD Accuri™ C6 Plus Personal Flow Cytometer (Becton Dickinson, USA) was used to observe cell apoptosis.

#### Tunel assay

DNA fragmentation was detected by TUNEL (Beyotime, Shanghai, China). KYSE30 cells were treated with different concentrations of the compounds for 12 hours. Cells were then fixed with 4 % paraformaldehyde for 30 min, discarded the paraformaldehyde and washed with PBS twice. Then 0.3 % triton X‐100 was added to permeabilize the cell membrane for 5 to 10 min. Washed twice with PBS, 50 uL of TUNEL staining solution (Tdt enzyme: fluorescent staining solution=5 μL:45 μL) was added to each well and incubated at 37 °C in a 5 % CO_2_ incubator in the dark for 1 hour. Fluorescence was observed under a fluorescence microscope (Eclipse Ti, Nikon, Japan).

#### Western Blot Assay

The protein was extracted after incubating the compounds with the KYSE30 for 48 hours. Cell lysate was prepared by using RIPA buffer with a PMSF ratio of 100 : 1. A total of 150 μL of lysate was added to a six‐well plate and incubated on ice for 30 minutes. The cells were then disrupted using a cell scraper and the lysed cell suspension was transferred to an EP tube. The tube was centrifuged at 12000 rpm for 10 minutes, and the supernatant containing the cellular proteins was collected. The proteins were then subjected to electrophoresis on an SDS‐PAGE gel and transferred to a polyvinylidene fluoride (PVDF) membrane. The membrane was blocked using a 5 % BSA blocking solution for 2 hours to prevent non‐specific binding. After washing with TBST solution, the membrane was incubated overnight at 4 °C with the primary antibody Stat3 (1 : 2000, CST), Smad2/3 (1 : 1000, CST), GAPDH (1 : 5000, Sangon Biotech), and cleaved caspase 3 (1 : 1000, CST). Following another wash with TBST, the membrane was incubated with an HRP‐conjugated secondary antibody at room temperature for 1 hour. Finally, Protein bands were visualized with an ECL luminescence solution.

#### Statistical Analysis

The results in this experiment are expressed as mean ±SD. Univariate analysis of variance (ANOVA) was used to assess the statistical significance of differences between groups. P<0.05 was considered to be statistically significant. All analyses were performed using Graphpad Prism 9.

## Conflict of interests

The authors declare no conflict of interest, financial or otherwise, in this paper.

1

## Supporting information

As a service to our authors and readers, this journal provides supporting information supplied by the authors. Such materials are peer reviewed and may be re‐organized for online delivery, but are not copy‐edited or typeset. Technical support issues arising from supporting information (other than missing files) should be addressed to the authors.

Supporting Information

## Data Availability

The data that support the findings of this study are available in the supplementary material of this article.
