# Supplementary material for: Synthesis of compounds based on the active domain of cabotegravir and their application in inhibiting tumor cells activity
Source: ChemistryOpen. 2024 Feb 5;13(7):e202300284. doi: 10.1002/open.202300284 (PMC11230920; doi:10.1002/open.202300284)
Supplement: Supplementary file 1 — Supporting Information [file OPEN-13-e202300284-s001.pdf]

ChemistryOpen

Supporting Information

Synthesis of compounds based on the active domain of cabotegravir and their application in inhibiting tumor cells activity

Ruyue Yang, Wenhui Yue, Dong Hu, Guidan Wang, Longfei Mao, Jiahe Huang, Huili Wang,* and Gaofeng Liang*

Supporting Information

Synthesis of compounds based on the active domain of cabotegravir and their application in inhibiting KYSE30 cell activity

Ruyue Yang [1], Wenhui Yue [1], Dong Hu [1], Guidan Wang [2], Longfei Mao [1], Jiahe Huang [1], Huili Wang [3,] *, Gaofeng Liang [1,] *

[1]*School of Basic Medicine and Forensic Medicine, Henan University of Science & Technology,*

Luoyang 471023, China;

[2] *School of Medical Technology and Engineering, Henan University of Science & Technology,*

Luoyang 471023, China;

[3]*University of North Carolina Hospitals, 101 Manning Dr, Chapel Hill, Orange County, NC 27599,*

USA

* Corresponding author:

Gao-Feng Liang, Ph.D.

Professor

School of Basic Medicine and Forensic Medicine, Henan University of Science & Technology,

263 kaiyuan Avenue, Luoyang 471000, China

Tel: +86-189-3901-5598, Fax: 86-0379-4830346

E-mail: lgfeng990448@163.com

Compound 3：^{1}H NMR (400 MHz, DMSO-d_6) δ 15.46 (d, *J* = 8.1 Hz, 1H), 8.75 (d, *J* = 3.2 Hz, 1H), 5.43 (d, *J* = 82.2 Hz, 1H), 4.87 (d, *J* = 80.4 Hz, 1H), 4.41 (d, *J* = 47.8 Hz, 1H), 4.14 (d, *J* = 53.8 Hz, 2H), 3.89 (d, *J* = 4.2 Hz, 3H), 3.14 (d, *J* = 85.1 Hz, 1H), 1.33 (d, *J* = 29.1 Hz, 3H).

Compound 4：^{1}H NMR (400 MHz, DMSO-d_6) δ 12.53 (s, 1H), 8.69 (s, 1H), 7.95 (s, 1H), 7.58 (s, 1H), 7.38 (s, 1H), 7.22 (s, 1H), 5.42 (d, *J* = 78.6 Hz, 1H), 4.87 (d, *J* = 76.4 Hz, 1H), 4.40 (d, *J* = 25.2 Hz, 1H), 4.14 (d, *J* = 68.8 Hz, 2H), 3.80 (d, *J* = 72.6 Hz, 3H), 3.13 (d, *J* = 67.9 Hz, 1H), 2.81 (d, *J* = 63.8 Hz, 1H), 1.29 (d, *J* = 48.7 Hz, 3H).

Compound 5a: ^{1}H NMR (600 MHz, DMSO-d_6): 12.55 (d, *J* = 6.0 Hz, 1H), 8.71 (s, 1H), 8.70 (d, *J* = 6.0 Hz, 1H), 8.13 (s, 1H), 7.76 (d, *J* = 6.0 Hz, 1H), 7.59 (d, *J* = 6.0 Hz, 1H), 7.45-7.34 (m, 6H), 5.65 (s, 2H), 5.53-5.33 (m, 1H), 4.96-4.77 (m, 1H), 4.45-4.36 (m, 1H), 4.18-4.05 (m, 2H), 3.91 (d, *J* = 6.0 Hz, 3H), 3.24-3.04 (m, 1H), 1.36-1.30 (m, 3H). ^{13}C NMR (150 MHz, DMSO-d_6): 174.16, 168.40, 162.21, 154.34, 151.99, 146.84, 144.20, 139.36, 136.46, 131.95, 131.62, 130.19, 129.30, 128.67, 128.45, 122.33, 121.18, 119.67, 118.03, 116.71, 82.74, 74.14, 60.97, 55.44, 53.55, 49.94, 18.39.

Calc. $C_{28}H_{26}N_6O_5$ $[M+H]^+$ m/z: 527.2043, found: 527.2031.

Compound 5b: ^{1}H NMR (600 MHz, DMSO-d_6): 12.55 (d, *J* = 6.0 Hz, 1H), 8.70 (d, *J* = 6.0 Hz, 1H), 8.67 (s, 1H), 8.14 (s, 1H), 7.76 (d, *J* = 12.0 Hz, 1H), 7.72 (d, *J* = 6.0 Hz, 1H), 7.61 (d, *J* = 6.0 Hz, 1H), 7.44 (t, J_1 = 6.0 Hz, J_2=6.0 Hz, 2H), 7.34 (t, J_1=6.0 Hz, J_2 = 6.0 Hz, 1H), 7.27 (d, *J* = 6.0 Hz, 1H), 5.75 (s, 2H), 5.53-5.34 (m, 1H), 4.96-4.78 (m, 1H), 4.45-4.36 (m, 1H), 4.17-4.04 (m, 2H), 3.91 (d, *J* = 6.0 Hz, 3H), 3.24-3.04 (m, 1H), 1.36-1.30 (m, 3H). ^{13}C NMR (150 MHz, DMSO-d_6): 174.09, 162.22, 154.34, 152.02, 146.66, 144.10, 139.37, 135.24, 133.42, 131.86, 131.06, 130.95, 130.19, 128.83, 123.39, 122.72, 121.25, 119.73, 118.16, 116.77, 82.27, 74.14, 60.98, 54.80, 53.63, 49.55, 18.39

Calc. $C_{28}H_{25}BrN_6O_5$ $[M+H]^+$ m/z: 605.1148, found: 605.1155.

Compound 5c: ^{1}H NMR(600 MHz, DMSO-d_6): 12.54 (d, *J* = 6.0 Hz, 1H), 8.70 (d, *J* = 6.0 Hz, 1H), 8.60 (s, 1H), 8.13 (s, 1H), 7.76 (d, *J* = 12.0 Hz, 1H), 7.60 (d, *J* = 6.0 Hz, 1H), 7.43 (t, J_1 = 6.0 Hz, J_2 = 6.0 Hz, 1H), 7.28-7.16 (m, 4H), 5.66 (s, 2H), 5.53-5.34

(m, 1H), 4.96-4.77 (m, 1H), 4.47-4.36 (m, 1H), 4.18-4.04 (m, 2H), 3.91 (d, *J* = 6.0 Hz, 3H), 3.24-3.04 (m, 1H), 2.36 (s, 3H), 1.36-1.30 (m, 3H). ^{13}C NMR (150 MHz, DMSO-d_6):174.15, 162.20, 154.33, 151.99, 146.71, 144.09, 139.35, 136.83, 134.51, 131.94, 130.93, 130.16, 129.26, 128.85, 126.80, 122.31, 121.20, 119.16, 118.16, 116.74, 82.73, 74.14, 60.97, 54.80, 51.65, 49.55, 31.78, 19.18, 18.39

Calc. $C_{29}H_{28}N_6O_5$ $[M+H]^+$ m/z: 541.2199, found: 541.2241.

Compound 5d: ^{1}H NMR(400 MHz, DMSO-d_6): 12.56 (d, *J* = 4.0 Hz, 1H), 8.75 (s, 1H), 8.70 (d, *J* = 4.0 Hz, 1H), 8.14 (s, 1H), 7.78 (t, J_1 = 4.0 Hz, J_2 = 8.0 Hz, 3H), 7.60-7.56 (m, 3H), 7.44 (t, J_1 = 8.0 Hz, J_2 = 4.0 Hz, 1H), 5.79 (s, 2H), 5.53-5.34 (m, 1H), 4.96-4.78 (m, 1H), 4.45-4.36 (m, 1H), 4.18-4.05 (m, 2H), 3.91-3.90 (m, 3H), 3.24-3.04 (m, 1H), 1.37-1.30 (m, 3H). ^{13}C NMR (100 MHz, DMSO-d_6): 174.16, 162.21, 154.33, 151.99, 146.96, 144.19, 144.08, 141.10, 139.38, 131.85, 131.62, 130.21, 129.15, 126.21, 122.63, 121.20, 119.73, 118.15, 118.03, 116.75, 82.74, 74.74, 60.97, 55.45, 52.88, 49.94, 18.39

Calc. $C_{29}H_{25}F_3N_6O_5$ $[M+H]^+$ m/z: 595.1917, found: 595.1928.

Compound 5e: ^{1}H NMR (600 MHz, DMSO-d_6): 12.55 (d, *J* = 6.0 Hz, 1H), 8.70 (d, *J* = 6.0 Hz, 2H), 8.15 (s, 1H), 7.85 (d, *J* = 12.0 Hz, 1H), 7.77 (d, *J* = 6.0 Hz, 1H), 7.72 (t, J_1 = 6.0 Hz, J_2 = 6.0 Hz, 1H), 7.61 (t, J_1 = 6.0 Hz, J_2 = 12.0 Hz, 2H), 7.44 (t, J_1 = 12.0 Hz, J_2 = 6.0 Hz, 1H), 7.28 (d, *J* = 6.0 Hz, 1H), 5.86 (s, 2H), 5.53-5.34 (m, 1H), 4.96-4.78 (m, 1H), 4.45-4.36 (m, 1H), 4.18-4.05 (m, 2H), 3.91 (d, *J* = 6.0 Hz, 3H), 3.24-3.04 (m, 1H), 1.36-1.30 (m, 3H). ^{13}C NMR (150 MHz, DMSO-d_6): 174.09, 162.22, 154.33, 151.99, 146.80, 144.20, 139.38, 134.05, 133.75, 131.81, 131.62, 131.49, 130.80, 130.19, 129.43, 127.19, 126.74, 125.56, 122.92, 121.25, 119.76, 118.15, 116.79, 82.74, 74.14, 60.97, 55.44, 50.25, 49.55, 18.41

Calc. $C_{29}H_{25}F_3N_6O_5$ $[M+H]^+$ m/z: 595.1917, found: 595.1943.

Compound 5f: ^{1}H NMR(600 MHz, DMSO-d_6): 12.55 (d, *J* = 6.0 Hz, 1H), 8.70-8.69 (m, 2H), 8.12 (s, 1H), 7.76 (d, *J* = 12.0 Hz, 1H), 7.57 (d, *J* = 6.0 Hz, 1H), 7.48-7.40 (m, 5H), 5.66 (s, 2H), 5.53-5.34 (m, 1H), 4.96-4.77 (m, 1H), 4.46-4.36 (m, 1H), 4.18-4.05 (m, 2H), 3.91 (d, *J* = 6.0 Hz, 3H), 3.24-3.04 (m, 1H), 1.37-1.30 (m, 3H). ^{13}C NMR (150 MHz, DMSO-d_6): 174.16, 162.22, 154.52, 152.02, 146.88, 144.20, 139.37, 135.42,

133.39, 131.89, 131.62, 130.43, 130.20, 129.30, 122.38, 121.18, 119.70, 118.16, 116.73, 82.74, 74.74, 60.98, 52.74, 49.55, 18.42

Calc. $C_{28}H_{25}ClN_6O_5$ $[M+H]^+$ m/z: 561.1653, found: 561.1699.

Compound 5g: ^{1}H NMR (600 MHz, DMSO-d_6): 12.55 (d, *J* = 6.0 Hz, 1H), 8.70-8.69 (m, 2H), 8.13 (s, 1H), 7.76 (d, *J* = 6.0 Hz, 1H), 7.59 (d, *J* = 6.0 Hz, 1H), 7.43 (t, J_1 = 12.0 Hz, J_2 = 6.0 Hz, 1H), 7.31 (t, J_1 = 12.0 Hz, J_2 = 6.0 Hz, 1H), 6.97 (s, 1H), 6.93-6.91 (m, 2H), 5.62 (s, 2H), 5.53-5.34 (m, 1H), 4.96-4.77 (m, 1H), 4.45-4.36 (m, 1H), 4.19-4.05 (m, 2H), 3.91 (d, *J* = 6.0 Hz, 3H), 3.76 (s, 3H), 3.24-3.04 (m, 1H), 1.36-1.30 (m, 3H). ^{13}C NMR (150 MHz, DMSO-d_6): 174.09, 162.21, 159.96, 154.34, 151.99, 146.82, 144.19, 139.36, 137.86, 131.95, 131.49, 130.46, 130.18, 122.33, 121.18, 120.53, 119.67, 118.16, 116.71, 114.27, 114.01, 82.74, 74.14, 60.97, 55.61, 53.48, 49.94, 18.39

Calc. $C_{29}H_{28}N_6O_6$ $[M+H]^+$ m/z: 557.2149, found: 557.2148.

Compound 5h: ^{1}H NMR (600 MHz, DMSO-d_6): 12.55 (d, *J* = 6.0 Hz, 1H), 8.70 (d, *J* = 6.0 Hz, 1H), 8.68 (s, 1H), 8.14 (s, 1H), 7.76 (d, *J* = 12.0 Hz, 1H), 7.61 (d, *J* = 6.0 Hz, 1H), 7.55 (d, *J* = 12.0 Hz, 1H), 7.45-7.39 (m, 3H), 7.32 (d, *J* = 12.0 Hz, 1H), 5.77 (s, 2H), 5.53-5.33 (m, 1H), 4.96-4.78 (m, 1H), 4.45-4.36 (m, 1H), 4.17-4.04 (m, 2H), 3.91 (d, *J* = 6.0 Hz, 3H), 3.24-3.04 (m, 1H), 1.36-1.30 (m, 3H). ^{13}C NMR (150 MHz, DMSO-d_6): 174.15, 162.21, 154.33, 152.01, 146.66, 144.20, 139.37, 133.63, 133.15, 131.86, 131.61, 131.10, 130.78, 130.14, 128.28, 122.68, 121.24, 119.72, 118.03, 116.76, 82.74, 74.14, 60.98, 55.44, 54.80, 51.30, 49.94, 18.39

Calc. $C_{28}H_{25}ClN_6O_5$ $[M+H]^+$ m/z: 561.1653, found: 561.1657.

Compound 5i: ^{1}H NMR (600 MHz, DMSO-d_6): 12.55 (d, *J* = 12.0 Hz, 1H), 8.74 (s, 1H), 8.70 (d, *J* = 6.0 Hz, 1H), 8.14 (s, 1H), 7.77 (d, *J* = 12.0 Hz, 1H), 7.60 (d, *J* = 6.0 Hz, 1H), 7.45-7.43 (m, 2H), 7.26-7.18 (m, 3H), 5.69 (s, 2H), 5.53-5.34 (m, 1H), 4.96-4.78 (m, 1H), 4.45-4.36 (m, 1H), 4.18-4.05 (m, 2H), 3.91 (d, *J* = 6.0 Hz, 3H), 3.24-3.04 (m, 1H), 1.36-1.30 (m, 3H). ^{13}C NMR (150 MHz, DMSO-d_6): 174.16, 163.46, 162.21, 154.50, 152.02, 146.90, 144.19, 139.37, 139.04, 131.88, 131.37, 130.19, 124.56, 122.47, 121.19, 119.71, 118.03, 116.74, 115.62, 115.46, 115.31, 82.27, 74.14, 60.97, 55.45, 52.86, 49.94, 18.41

Calc. $C_{28}H_{25}FN_6O_5$ $[M+H]^+$ m/z: 545.1949, found: 545.1937.

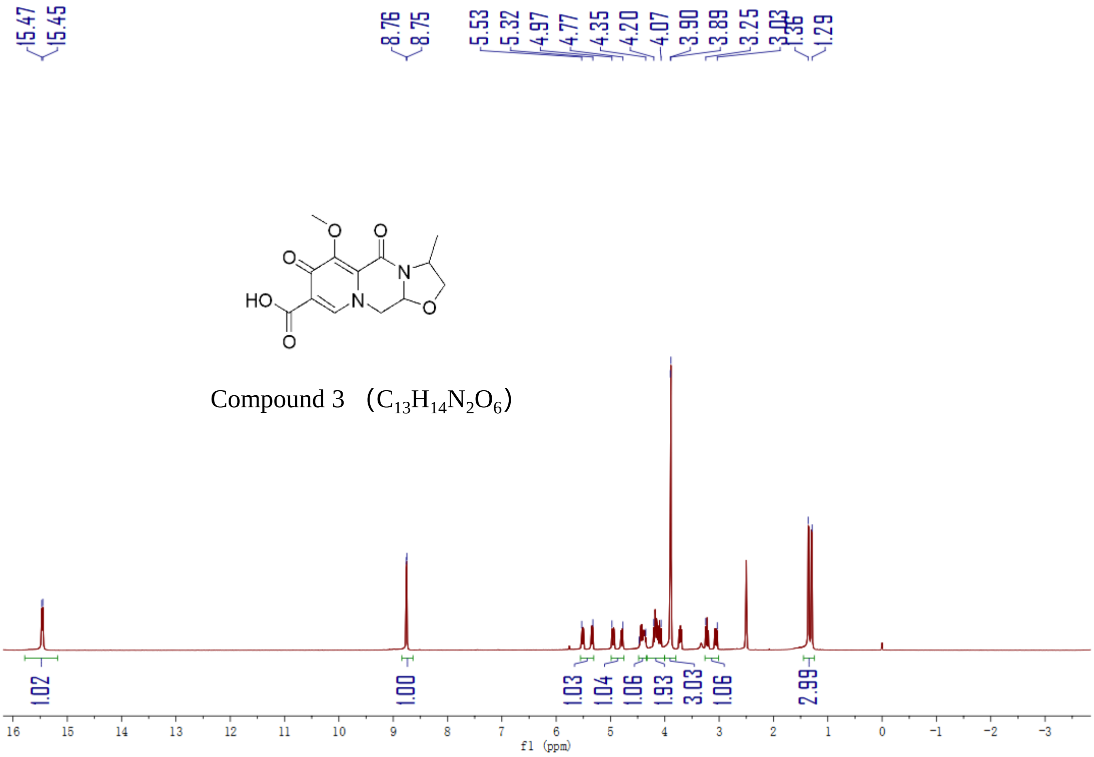

Figure S1: ^{1}H NMR spectrum of compound 3

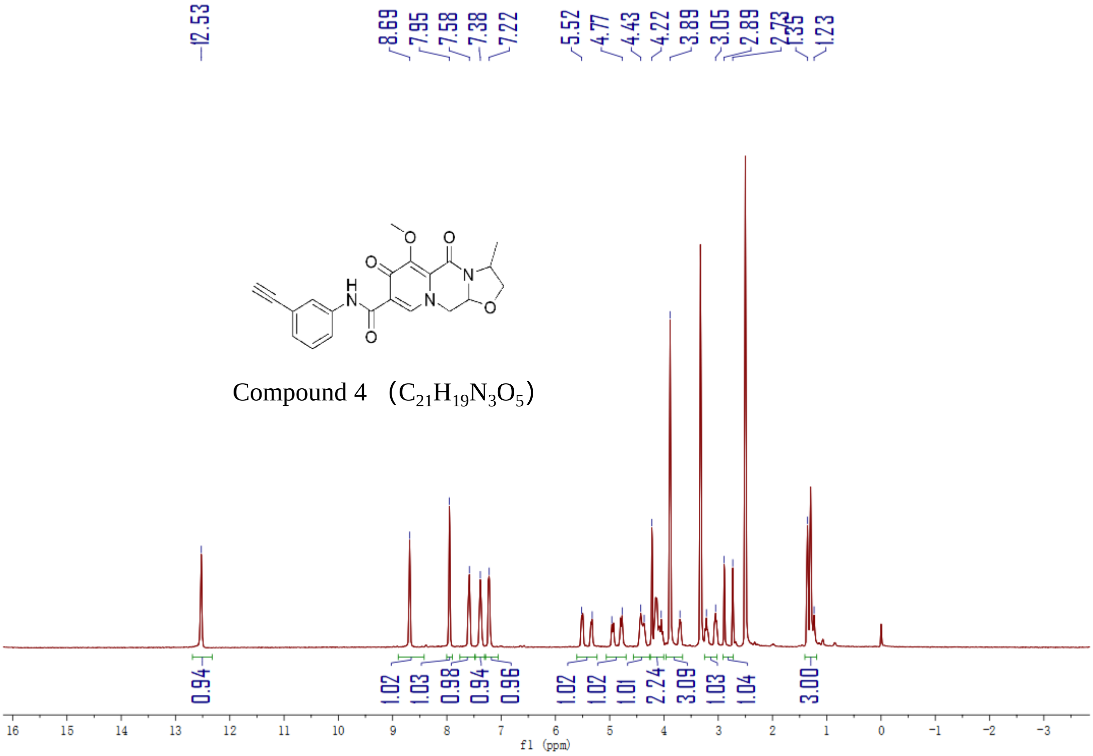

Figure S2: ^{1}H NMR spectrum of compound 4

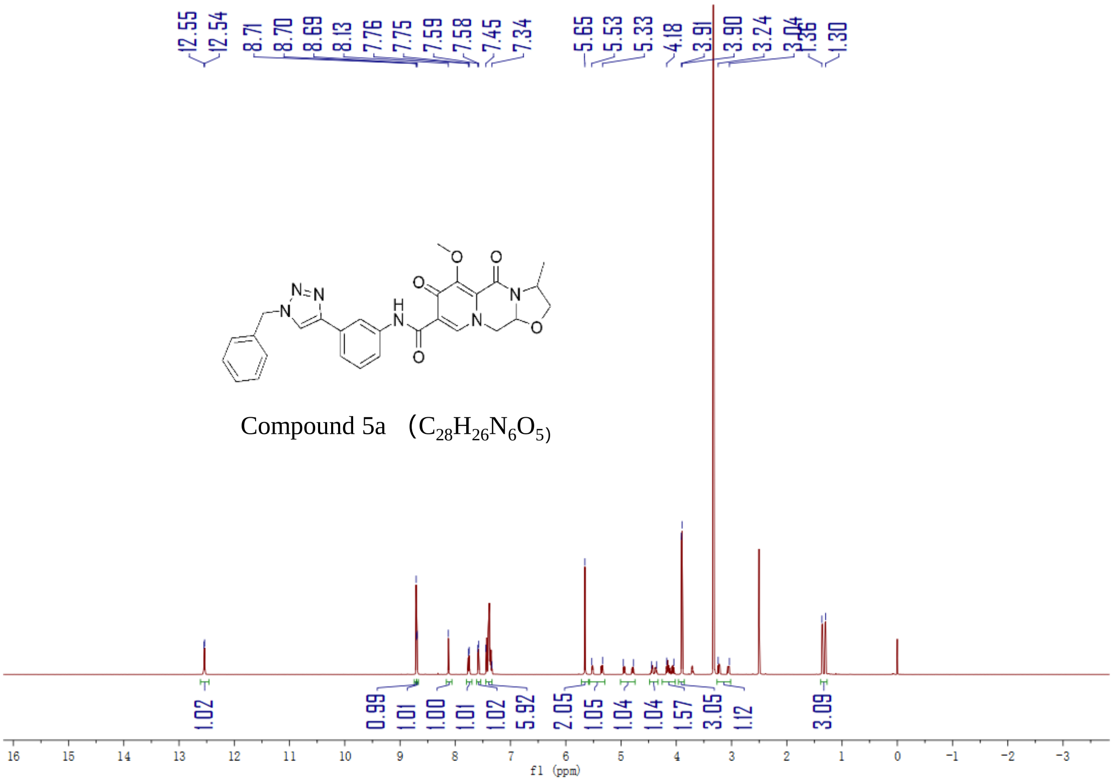

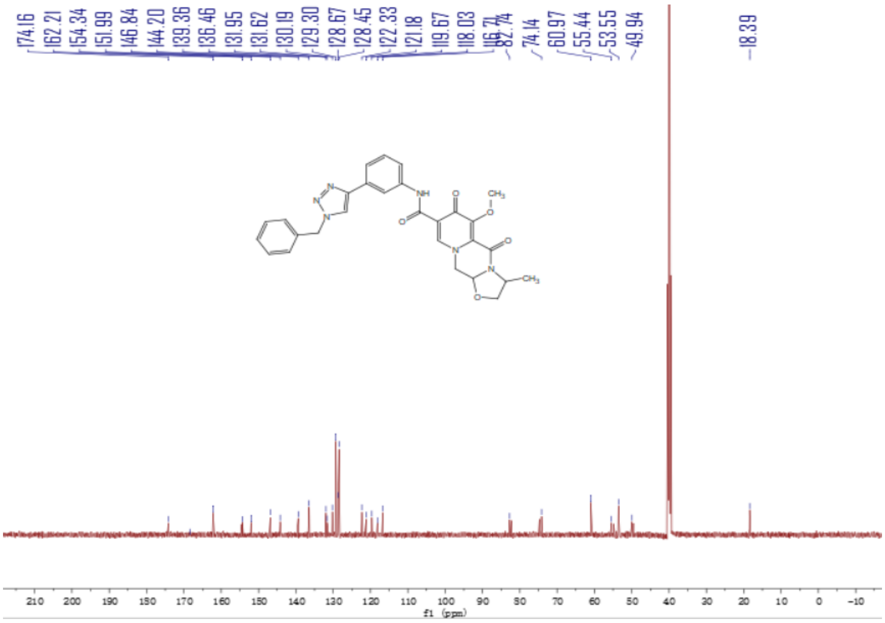

Figure S3. ^{1}H NMR and ^{13}C NMR spectrums of compound 5a

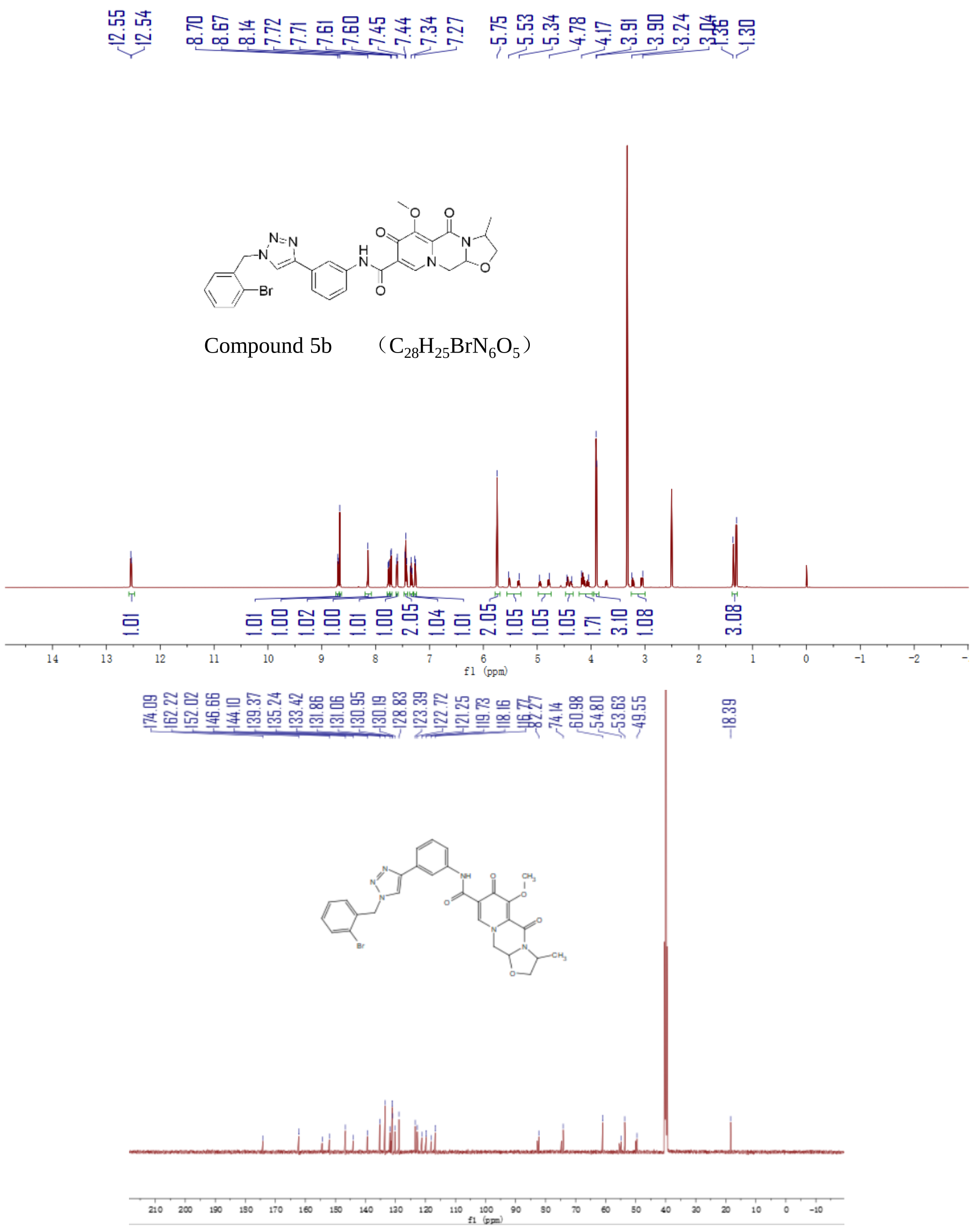

Figure S4. ^{1}H NMR and ^{13}C NMR spectrums of compound 5b

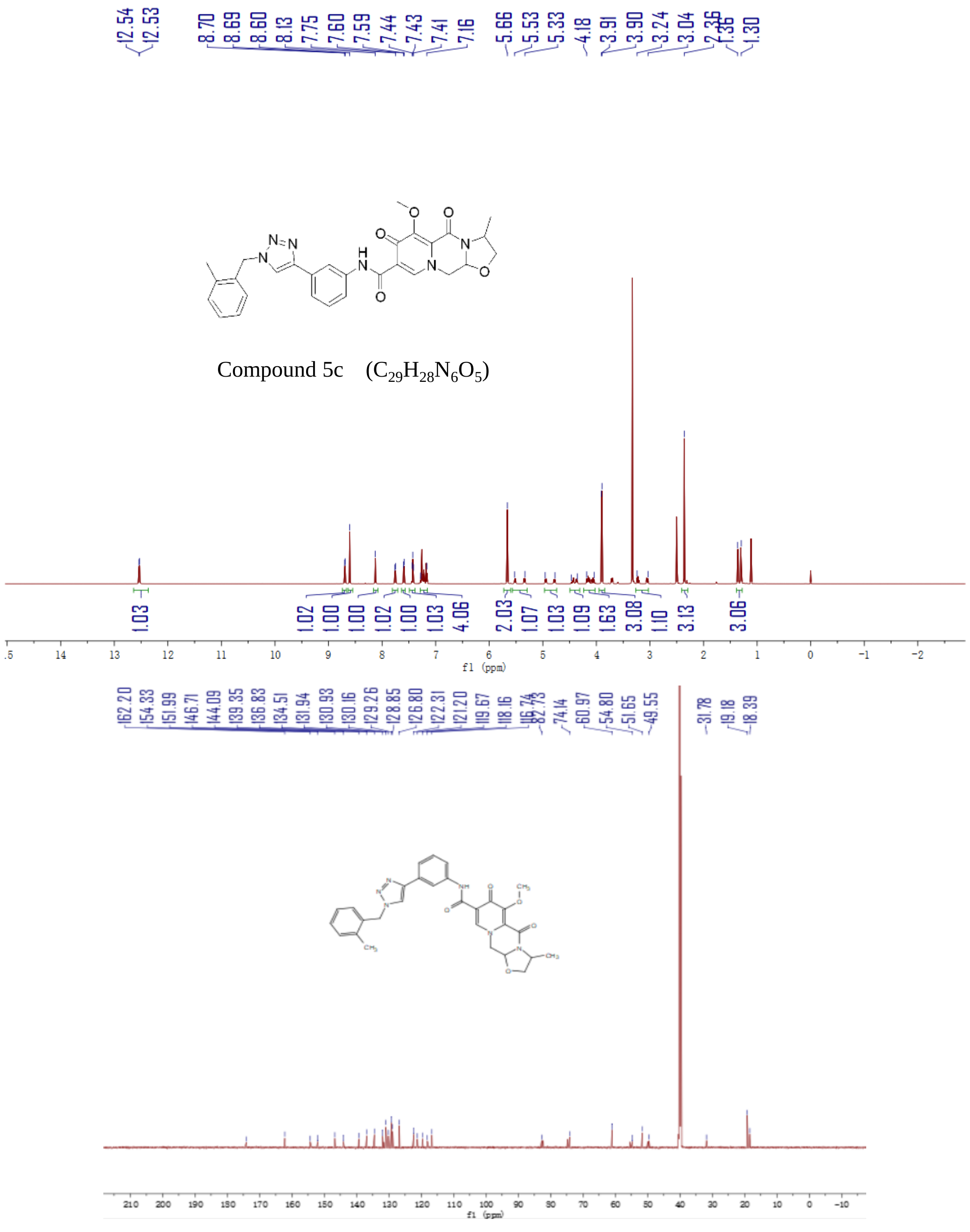

Figure S5. ^{1}H NMR and ^{13}C NMR spectrums of compound 5c

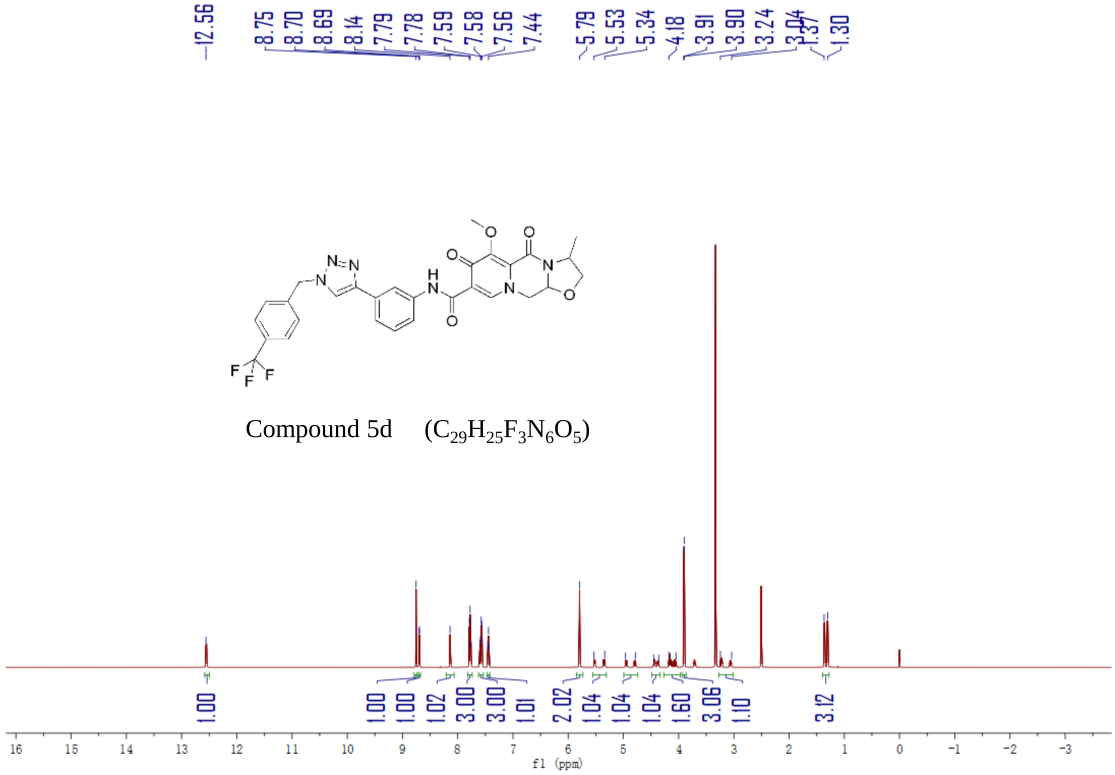

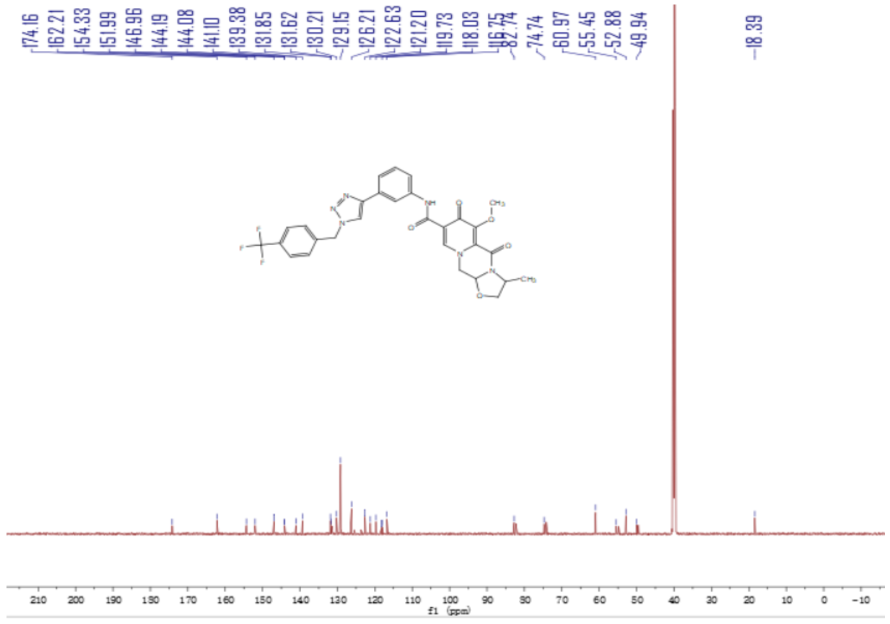

Figure S6. ^{1}H NMR and ^{13}C NMR spectrums of compound 5d

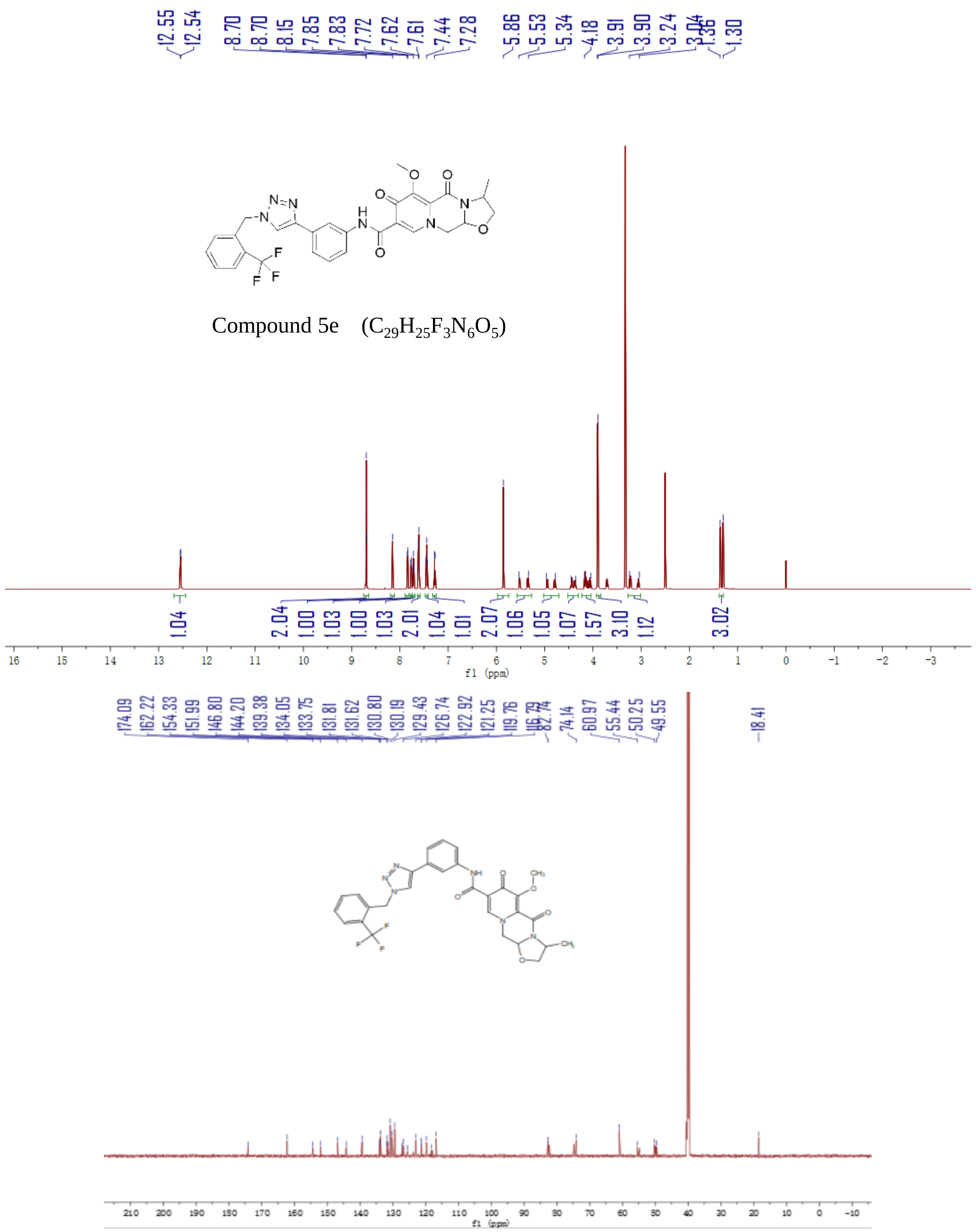

Figure S7. ^{1}H NMR and ^{13}C NMR spectrums of compound 5e

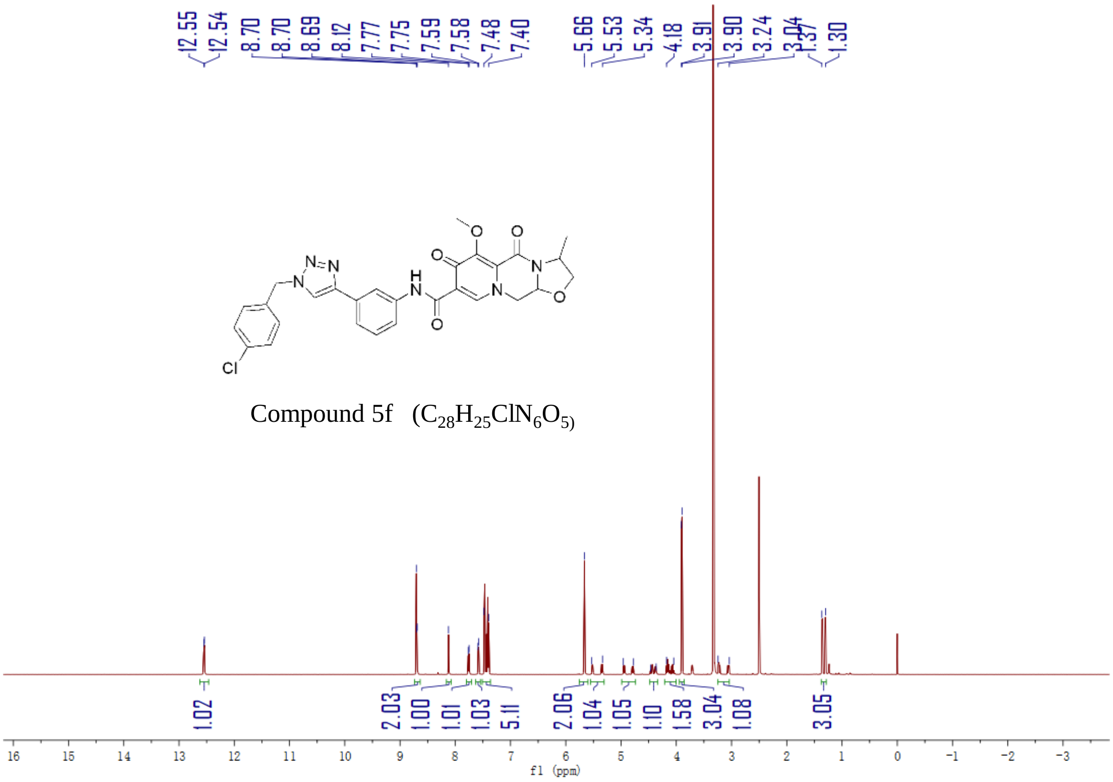

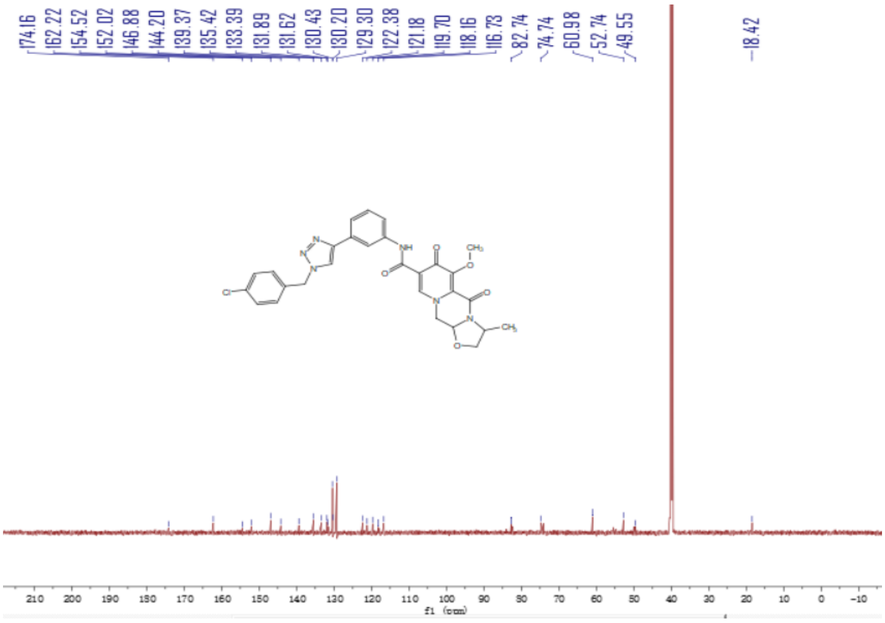

Figure S8. ^{1}H NMR and ^{13}C NMR spectrums of compound 5f

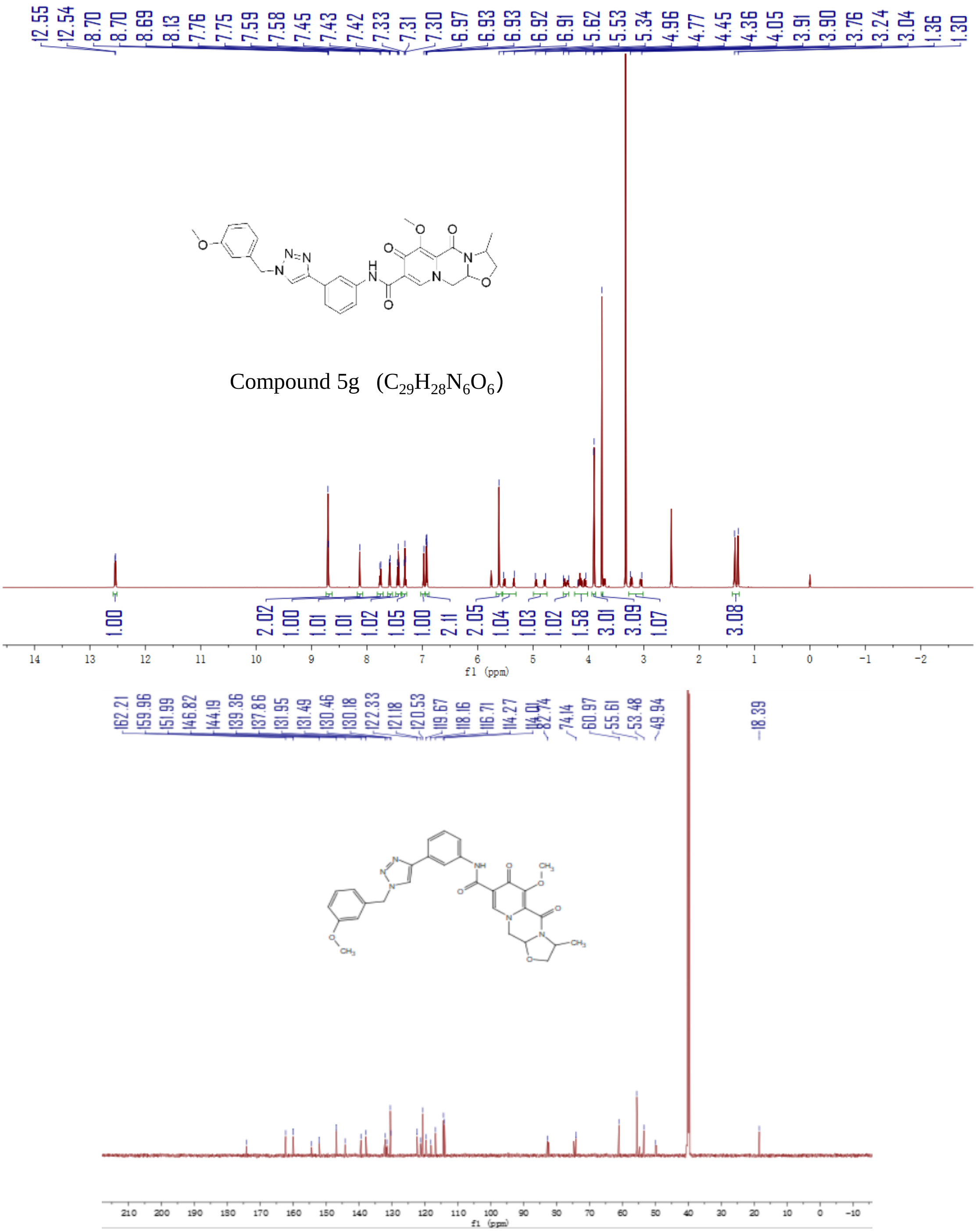

Figure S9. ^{1}H NMR and ^{13}C NMR spectrums of compound 5g

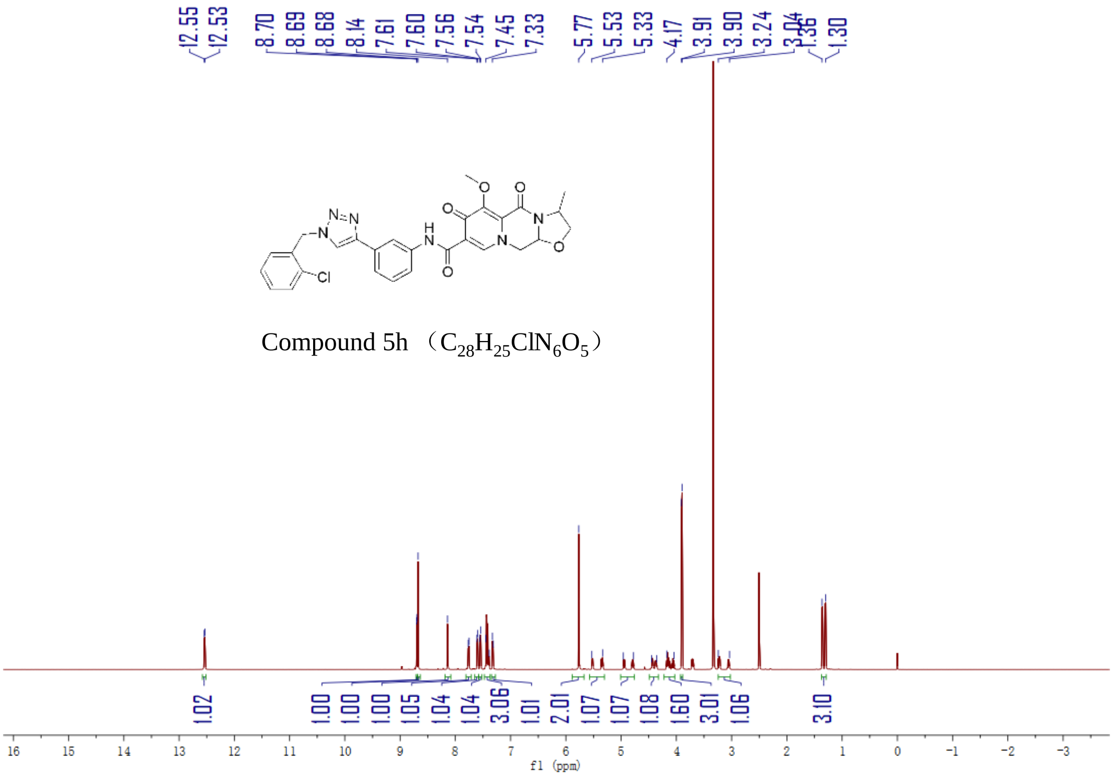

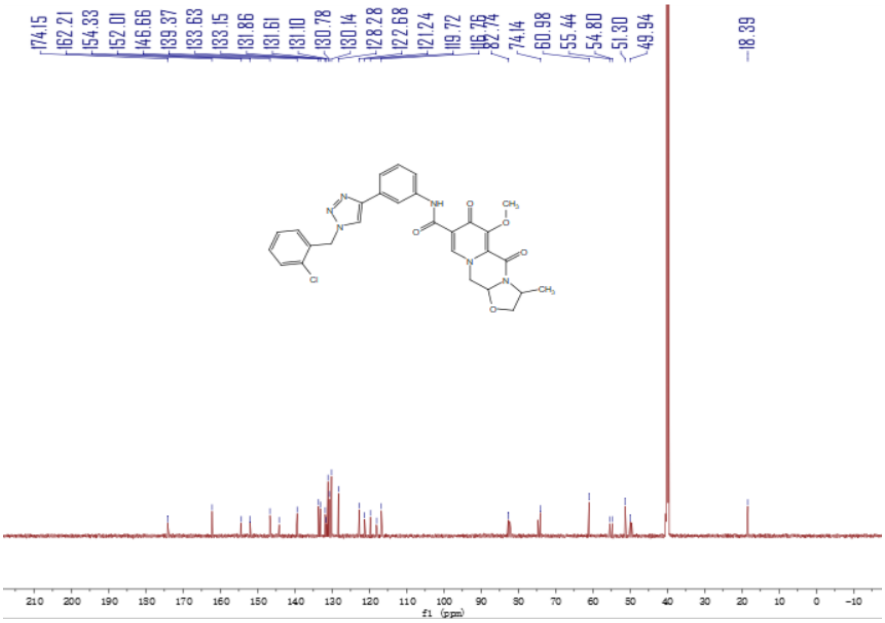

Figure S10. ^{1}H NMR and ^{13}C NMR spectrums of compound 5h

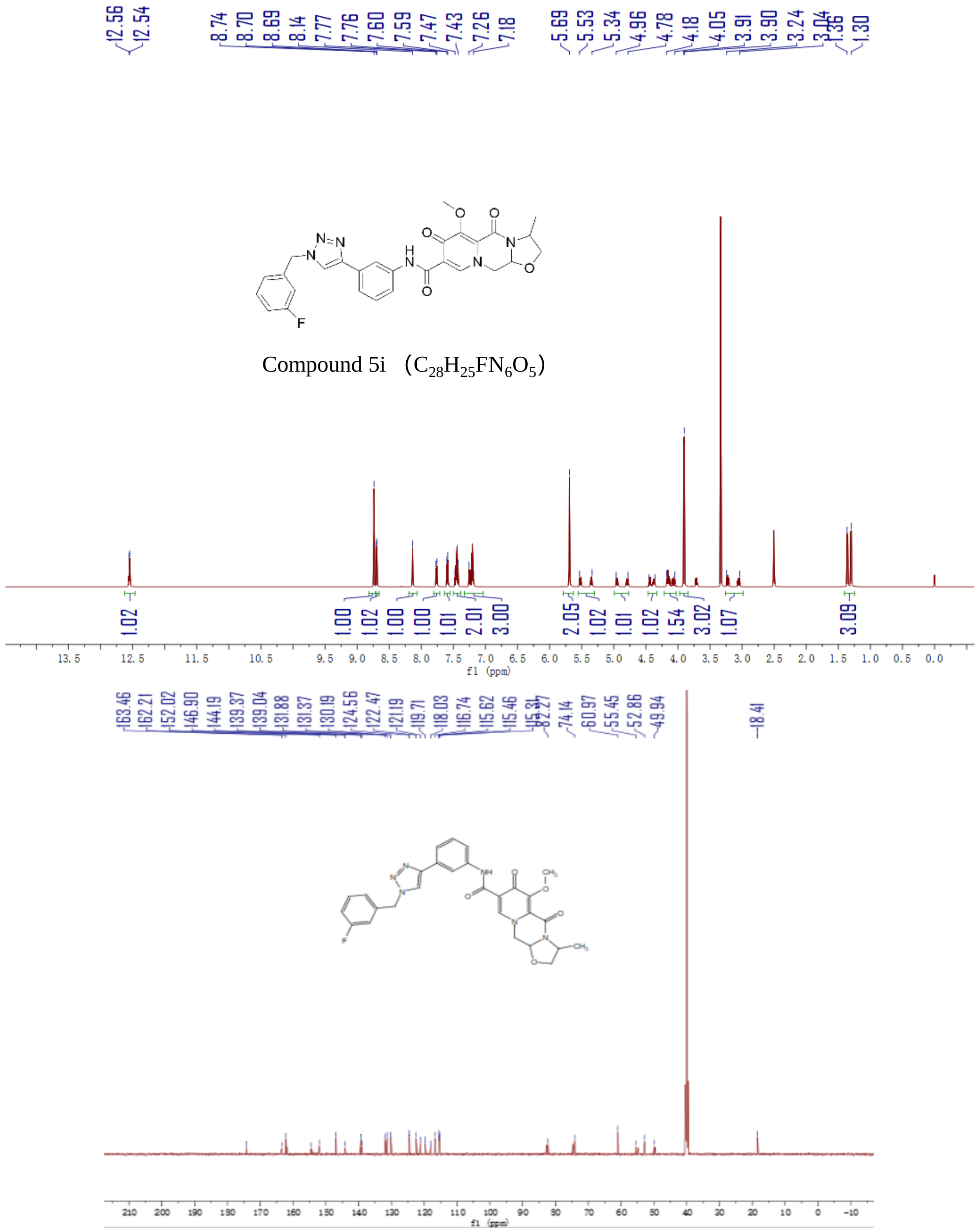

Figure S11. ^{1}H NMR and ^{13}C NMR spectrums of compound 5i

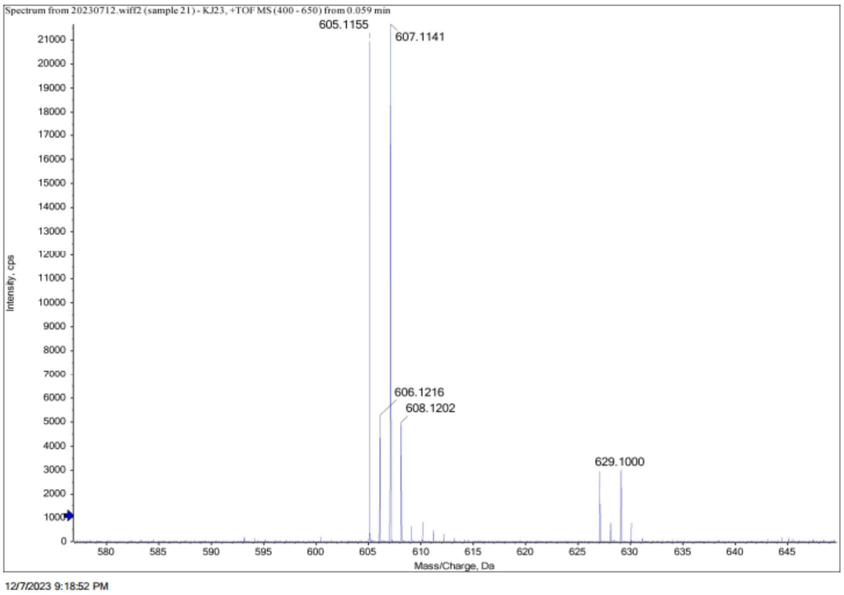

Figure S12. Mass spectrum of compound 5b

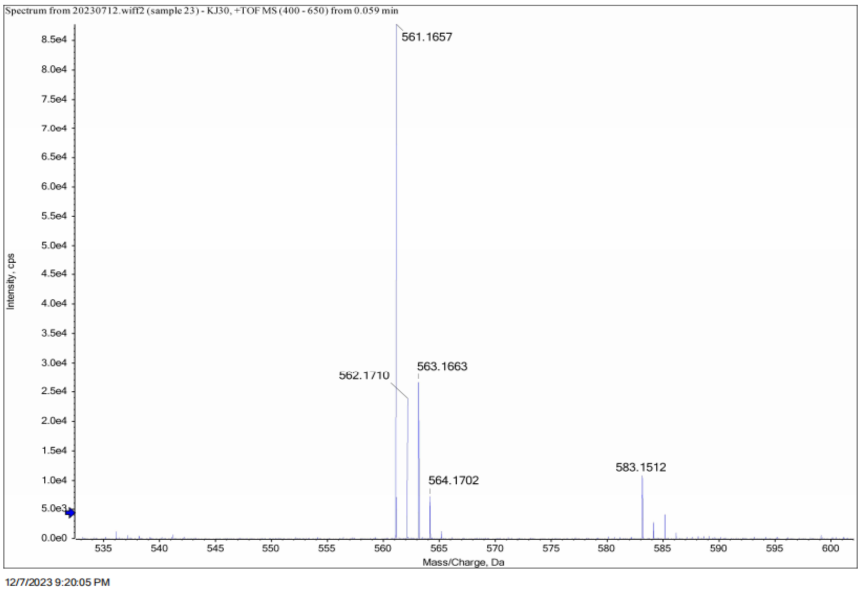

Figure S13: Mass spectrum of compound 5h
